# The Dielectrophoretic Interactions of Curved Particles in a DC Electric Field

**DOI:** 10.3390/mi16050596

**Published:** 2025-05-20

**Authors:** Zhiwei Huang, Tong Zhang, Jing Feng, Yage Wang

**Affiliations:** Mechanical and Electrical Engineering College, Guangdong University of Science and Technology, Dongguan 523668, China

**Keywords:** dielectrophoretic, deformation, curved particles, microfluidics

## Abstract

In practical dielectrophoretic cell interaction experiments, cells do not always exhibit circular or rod-like shapes, making the study of dielectrophoretic interactions among irregularly shaped particles of significant importance. We established a mathematical model for curved particles to analyze their mutual dielectrophoretic interactions, incorporating particle deformability by varying their shear modulus, and employed the arbitrary Lagrangian–Eulerian method to describe particle motion and deformation. The results demonstrate that under the influence of a direct current electric field, curved particles undergo rotation, deformation, and mutual attraction due to dielectrophoresis, eventually forming a stable alignment parallel to the applied electric field. Adjusting the electric field strength effectively modulates the interaction intensity and movement velocity between particles. This study elucidates the fundamental principles governing dielectrophoretic interactions among deformable curved particles in DC electric fields, providing theoretical guidance for dielectrophoretic manipulation experiments involving biological cells, metallic particles, and other entities under DC electric fields.

## 1. Introduction

Dielectrophoresis (DEP) is a manipulation technique based on the polarization of particles induced by non-uniform electric fields, which has been widely applied for the separation, trapping, alignment, and assembly of micro-/nanoscale particles [1,2,3,4]. The principle is that under the action of a non-uniform electric field, neutral particles experience DEP forces due to the differences in polarization properties between the particles and the surrounding medium. This enables label-free and high-precision particle manipulation [5,6,7,8]. The introduction of multiple micron particles into a uniform electric field induces localized field inhomogeneity, thereby subjecting the particles to DEP forces. This dielectrophoretic effect arising from the presence of multiple particles is commonly referred to as dielectrophoretic interactions [9,10,11,12,13]. When interparticle interactions are taken into consideration, the influence of particles on the surrounding electric field cannot be neglected, rendering the conventional electric dipole approximation inapplicable in such scenarios [14,15].

In theoretical studies, biological cells are generally regarded as particles with distinct electrical properties, where parameters such as the dielectric constant, conductivity, and size exhibit variations [16,17,18,19,20]. Numerous prior DEP investigations have been conducted based on rigid particles, neglecting particle deformation [21]. The interactions induced by particle deformation differ from those of rigid particles and more accurately represent real cellular behavior [12,22]. Furthermore, although simplifying cells or bacteria as spherical or rod-shaped structures can facilitate theoretical calculations to some extent, certain biological cells deviate from such regular geometries. For instance, *Vibrio cholerae* and *Campylobacter* species exhibit curved morphologies [23,24]. This observation holds significant practical implications for studying interactions among irregularly shaped particles [25]. For instance, there exists a significant disparity in the dielectrophoretic interaction mechanisms between spindle-shaped rigid particles and rod-shaped deformable particles [14,26]. Unlike scenarios where only DEP is at play, when particles are subjected to the combined effects of DEP and induced-charge electrophoresis, the repulsion or attraction between particles depends on structural design [27]. Guided by theoretical insights, dielectrophoretic particle manipulation has numerous practical applications. For instance, through microchannel design, liquid biopsy can be achieved without requiring focusing, thereby reducing system complexity [28]. Lab-on-a-chip systems constructed using fully integrated circuits for dielectrophoretic control enable highly precise regulation of dielectrophoretic parameters [29]. Furthermore, DEP technology has demonstrated capabilities in antimicrobial resistance diagnostics and lab-on-a-chip applications, achieving label-free separation of live and dead bacteria while showing correlations with label-free AMR estimation [30,31]. The manipulation of particles or droplets based on optically induced dielectrophoresis (ODEP) is also a research hotspot. For example, intelligent droplet control can be achieved by integrating ODEP with image recognition [32]. In such processes, droplet shapes are not limited to spheres or rods, necessitating more in-depth studies on dielectrophoretic interactions involving irregularly shaped particles. This highlights the need to explore how non-spherical geometries influence electric field distributions and particle dynamics, particularly for applications requiring precision in microfluidics or biomedical engineering.

In this study, the dielectrophoretic interaction between two curved deformable particles is systematically investigated through numerical simulation. The DEP effect induces particle motion, deformation, and mutual interaction under an applied direct current (DC) electric field. The deformability of particles is characterized by their shear modulus. A mathematical model integrating the electric field, flow field, and deformable particles is established based on the arbitrary Lagrangian–Eulerian (ALE) method and the thin electric double-layer (EDL) assumption. The influences of the electric field intensity, particle arrangement, size, and shear modulus on particle deformation and motion are analyzed in detail. The results demonstrate that particles undergo rotation, deformation, and mutual attraction under the electric field, eventually aligning parallel to the field direction. These findings are expected to provide theoretical guidance for the manipulation of biological cells and micron particles.

## 2. Materials and Methods

### 2.1. Mathematical Model

**Figure 1:**
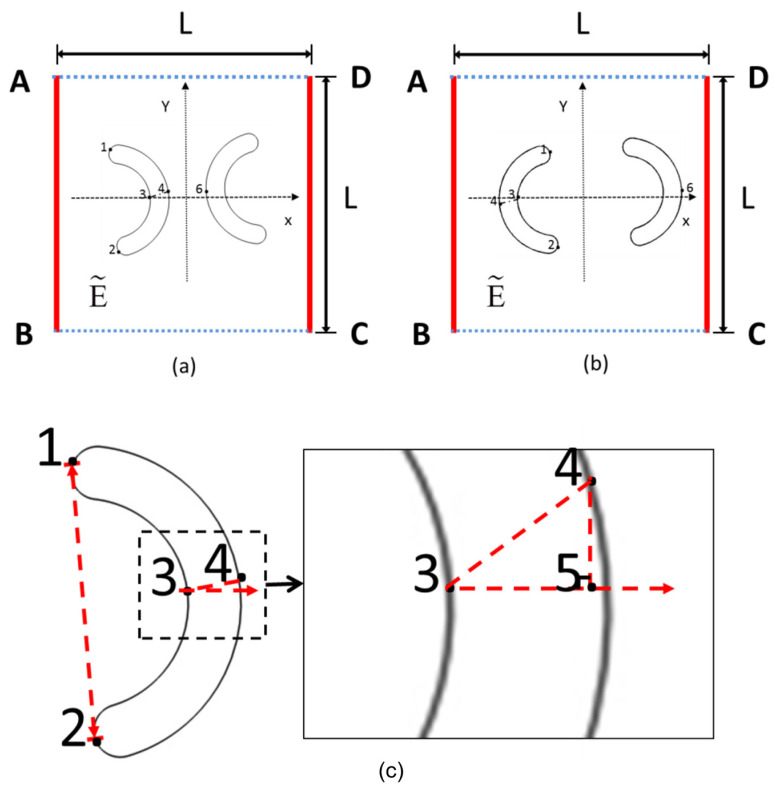
Schematic diagram of the model. (**a**) Back-to-back curved particles arrangement, (**b**) face-to-face particle arrangement, (**c**) characteristic calculation points of particles. Points 1 and 2 mark the upper and lower endpoints, Points 3 and 4 denote the mid-points of the outer and inner circular arcs, and Point 5 is determined by the intersection of a horizontal ray extending from Point 3 and a vertical line descending from Point 4.

Figure 1 presents the two-dimensional structural model of this study, featuring two curved particles suspended within a square fluid domain. These curved particles are constructed by overlapping two semicircles, with outer and inner circle diameters of 30 μm and 20 μm, respectively. The center coordinates of the left and right semicircles are (−20, 0) and (20, 0), respectively. A fillet radius of 2.5 μm is applied at the junction between the two semicircles. To facilitate the analysis of particle rotation and deformation, five characteristic points are selected on each curved particle: Points 1 and 2 mark the upper and lower endpoints, Points 3 and 4 denote the midpoints of the outer and inner circular arcs, and Point 5 is determined by the intersection of a horizontal ray extending from Point 3 and a vertical line descending from Point 4. The deformation and rotation of the curved particles are analyzed by tracking the coordinate variations in these characteristic points. The square fluid domain has a side length of 400 μm, with a two-dimensional coordinate system established at its center. Initially, the two curved particles are rotated by 5°, with the angles between edges 3-4 and 3-5 also set at 5°. In Figure 1a, the concave sides of the particles face toward the coordinate center, while in Figure 1b, they face outward, away from the center. For clarity in discussion, the configuration in Figure 1a is referred to as “back-to-back” arrangement, and that in Figure 1b as “face-to-face” arrangement.

Figure 1c displays the contours of back-to-back arcuate curved particles along with their characteristic points (the selection method for characteristic points in face-to-face curved particles is identical to that of back-to-back curved particles). By calculating the coordinates of these characteristic points, the deformation and rotation of the curved particles can be determined using the following equations:

(1) $$L_{12}=\sqrt{{\left(x_{1}^{*}-x_{2}^{*}\right)}^{2}+{\left(y_{1}^{*}-y_{2}^{*}\right)}^{2}}$$

(2) $$L_{34}=\sqrt{{\left(x_{3}^{*}-x_{4}^{*}\right)}^{2}+{\left(y_{3}^{*}-y_{4}^{*}\right)}^{2}}$$

(3) $$L_{35}=\left|x_{3}^{*}-x_{4}^{*}\right|$$

(4) $$\cos\theta =\frac{L_{35}}{L_{34}}$$

where $x_{i}^{*}$ and $y_{i}^{*}$ denote the coordinate values of the corresponding points, respectively, and θ represents the rotation angle of the particle. The * indicates that the data have undergone dimensionless processing.

### 2.2. Boundary Conditions

In this study, the self-weight of the curved particles is neglected; thus, the density of the curved particles is set equal to that of the fluid in the flow field. A uniform electric field is generated within the square fluid domain by applying an electric potential through electrodes positioned at its boundaries. The characteristic dimension of the curved particles is one order of magnitude smaller than that of the square fluid domain; therefore, based on the thin EDL assumption, the net charge within the EDL is considered zero [33]. An electric potential $\phi =\varphi_{0}$ is applied to boundary AB, while boundary DC is subjected to an electric potential ϕ=0. Boundaries AD and BC are electrically insulated.

The electric field is characterized by the Laplace equation [14]:

(5) $${𝛻}^{2}\phi =0$$

(6) E=−𝛻ϕ

(7) n⋅𝛻ϕ=0

In a square domain, the flow field boundaries AB and DC are defined as open boundaries, while AD and BC are designated as symmetry boundaries. Open boundaries represent interfaces that interact with extensive fluid reservoirs, characterized by the presence of normal stress and the absence of viscous stress. Symmetry boundaries denote impermeable surfaces with zero shear stress, where the no-slip condition is imposed such that the fluid velocity relative to the wall velocity vanishes [14].

The flow field is governed by the continuity equation and the Navier–Stokes equations [34,35]:

(8) 𝛻⋅u=0

(9) $$\rho_{f}\frac{\partial u}{\partial t}=𝛻\cdot \left[-pI+\mu \left(𝛻u+𝛻u^{T}\right)\right]$$

Here, $\rho_{f}$, pI, u, and μ represent the fluid density, fluid pressure term, fluid velocity, and dynamic viscosity, respectively.

The electroosmotic flow generated by the fluid and particles is relatively weak, with its velocity being negligible compared to the DEP velocity of the particles. Therefore, a no-slip boundary condition is applied to the particle surfaces. The particle velocity is obtained through the following equation [36]:

(10) $$u_{pi}=\frac{\varepsilon_{f}\zeta_{pi}}{\mu }\left(I-nn\right)\cdot 𝛻\phi +\frac{\partial S}{\partial t}$$

(11) $$\rho_{p}\frac{\partial^{2}S}{\partial t^{2}}-𝛻\cdot \sigma \left(S\right)=0$$

The right-hand side of Equation (10) consists of two components, the first of which represents the electrophoretic velocity of the particles. In this study, since the particles are electrically neutral, their conductivity and dielectric permittivity are not considered, and their surface zeta potential is zero. Consequently, this velocity component equals zero. The latter part represents the DEP velocity of the particle, where ***S*** denotes the DEP displacement of the particle. $\rho_{p}$ corresponds to the particle density, while $\sigma \left(S\right)$ signifies the Cauchy stress of the particle.

The deformation of particles can be characterized by the neo-Hookean model [14]:

(12) $$W=\frac{G_{0}}{2}\left(I_{c}-3\right)+\frac{1}{D}{\left(J-1\right)}^{2}$$

Here, $G_{0}$ represents the shear modulus of the material, $I_{c}$ denotes the invariant of the Cauchy–Green tensor, and J stands for the incompressibility parameter of the material.

The dielectrophoretic force acting on the particle is expressed as follows [13]:

(13) $$F_{dep}=2\pi \varepsilon_{f}a^{3}Re[k\;(\omega )]𝛻({\left|E\right|}^{2})$$

where a is the particle radius. $k\left(\omega \right)=(\varepsilon_{p}^{*}-{\tilde{\varepsilon }}_{f}^{*})/\left(2\varepsilon_{f}^{*}+\varepsilon_{P}^{*}\right)$ is the Clausius–Mossotti factor. Re⁡[k ⁡(ω)] represents the real part of the complex Clausius–Mossotti factor, and a negative (positive) value represents a negative (positive) DEP motion, in which particles are pushed to the region with a lower (higher) electric field strength. In this study, Re⁡[k ⁡(ω)] is positive, so the particles experience a positive dielectrophoretic force and attract each other.

The aforementioned mathematical model is solved using COMSOL Multiphysics 5.3, wherein particle motion and deformation are characterized via the ALE method [37,38]. Furthermore, when the model is implemented with identical parameters and compared with that of Ai et al. [21], the results demonstrate favorable agreement, thereby validating the model’s accuracy (Figure S1).

## 3. Results and Discussion

### 3.1. Electric Field Intensity

**Figure 2:**
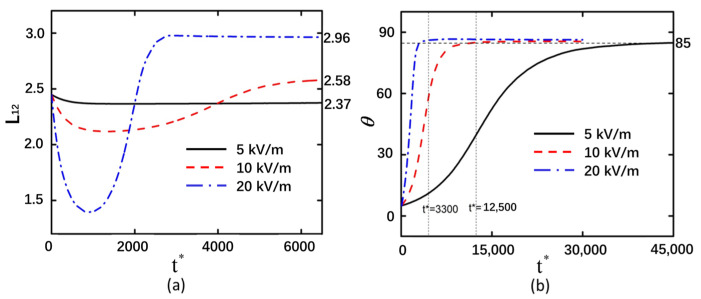
Back-to-back arrangement of curved particles with shear modulus of 20 Pa under electric field strengths of 5 kV/m, 10 kV/m, and 20 kV/m. (**a**) Deformation of the left curved particle. (**b**) Rotation angle of the left curved particle.

The electric field intensity exerts a significant influence on the motion of particles subjected to DEP. In an electric field, back-to-back curved particles undergo deformation over time under the influence of electric field forces, initially experiencing compressive bending followed by tensile (Figure 2a). Under the same shear modulus, higher electric field intensities result in greater degrees of compression and tensile of the curved particles, leading to increased deformation magnitudes. Under an electric field intensity of 5 kV/m, the deformation of the curved particles was measured to be 0.073. When the electric field intensity was increased to 10 kV/m, the corresponding deformation reached 0.13. Further elevation of the electric field intensity to 20 kV/m resulted in a more pronounced deformation of 0.51 in the curved particles. The greater the electric field intensity, the shorter the time required for the particle to rotate to 85° (Figure 2b).

The curved particle is not equivalent to a uniform spherical particle, as the differential polarization extent across its entire structure results in a non-uniform distribution of the electric field intensity in the vicinity of the curved particle. The electric field intensity is higher on the upper and lower outer sides of both ends of the curved particle, causing the ends to move toward regions of lower electric field intensity. This phenomenon is characterized as the compression of the curved particle (Figure S2). The electric field on the outer sides of the two curved particles exhibits a symmetric distribution. The electric field intensity at the lower end of the left particle is equal to that at the upper end of the right particle, both of which are stronger than the intensities at the upper end of the left particle and the lower end of the right particle. This asymmetric field distribution induces a non-equilibrium state, resulting in rotational motion of the curved particles. As the particles rotate, the orientation of the applied electric field acting upon them changes accordingly. Consequently, the electric field intensities at the extremities of the curved particles undergo corresponding modifications. Under a direct current electric field, both rod-shaped deformable particles initially experience compressive stress at their ends and tensile stress along their lateral surfaces during the deformation phase. As rotation proceeds, the stress state transitions from compressive at the ends to tensile, which is consistent with the findings of this study [14]. In this study, only the DEP force is considered. The generation of DEP force on particles is associated with the accumulation of charges at the particle interface under the influence of an external electric field. As the particle rotates, the distribution of surface charges changes, leading to an alteration in the direction of the DEP force. Consequently, the particle transitions from a compressed state to a stretched state. The applied electric field not only influences the rotation of the curved particle but also concurrently affects its deformation. When the electric field strength is higher on the outer side of the curved particle, it compresses the particle toward the region with lower electric field strength.

The electric field intensity is stronger at the outer regions of both ends of the curved particles. As the ends of the curved particles are compressed toward the center, the electric field intensity remains constant. When the deformation of the curved particle ends reaches a certain threshold, further compression toward the center ceases, and the deformation stabilizes. At this stage, the DEP force is insufficient to overcome the elastic resistance of the curved particles (Figure S2). The electric field intensity near the two ends of the curved particle exhibits minimal disparity compared to that in regions distal to the particle, resulting in negligible rotational motion of the particle at t* = 2000. The relatively low electric field strength at this stage yields insufficient DEP force to alter the orientation of the curved particle. Non-uniform distribution of the electric field is observed across the surface of the curved particle, with enhanced field intensity at the extremities compared to the central region. This field gradient induces continuous rotation of the particle. As two curved particles approach each other, mutual interaction forces between them generate counteracting torques that progressively inhibit rotational motion. This equilibrium state arises from the balanced yet oppositely directed forces acting on both particles.

When the applied electric field intensity is high, the electric field strength around the curved particles is also elevated, resulting in greater deformation of the curved particles and a faster rate of change in their rotational angle. In Figure 2a, the points on the Y-axis where the red dashed line and blue dash–dotted line ultimately stabilize are larger than their initial Y-axis values. This indicates that the curved particles undergo net stretching after the electric field is applied, with the magnitude of stretching increasing proportionally to the electric field intensity.

**Figure 3:**
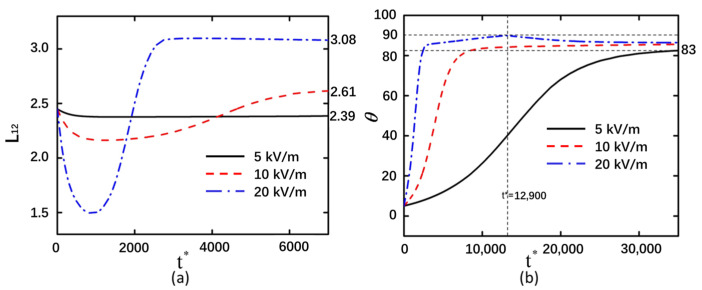
Face-to-face arrangement of curved particles with shear modulus of 20 Pa under electric field strengths of 5 kV/m, 10 kV/m, and 20 kV/m. (**a**) Deformation of the left curved particle. (**b**) Rotation angle of the left curved particle.

The variation trends of the deformation and rotation angle with time are consistent between back-to-back and face-to-face particle arrangements, with the deformation of back-to-back particles being slightly greater than that of face-to-face particles (Figure 3). The electric field intensity significantly influences the final arrangement of face-to-face aligned particles. When the electric field strength is 5 kV/m, curved particles remain incapable of converging even at t* = 200,000. Under identical temporal conditions, particles subjected to a 10 kV/m field achieve cross-type convergence. At a higher field intensity of 20 kV/m, the morphological arrangement of curved particles exhibits complete consistency with back-to-back alignment by t* = 30,000. Under an applied electric field intensity of 5 kV/m, the electric field strength between two curved particles is relatively weak. Due to the non-uniform geometry of the curved particles, polarization heterogeneity occurs, resulting in stronger electric field concentrations at the particle extremities (Figure S3). The ends experiencing greater electric field forces are consequently driven toward regions of lower field intensity, inducing a rotational phenomenon analogous to that observed in back-to-back particle configurations. The asymmetric field distribution across the curved particle terminals generates differential DEP forces, thereby prompting particle rotation centered on the weakened field region at the particle midpoint. Furthermore, minimal deformation differences were observed between face-to-face and back-to-back curved particle arrangements, indicating that spatial orientation does not significantly influence particle deformation magnitude.

At an electric field intensity of 20 kV/m, it was observed that the particle rotation reached 90° at t* = 12,900. Under these conditions, the two curved particles rotated and became elongated, with no interaction between them along the Y-direction. The electric field intensity exhibited a symmetrical distribution between the left and right curved particles (Figure S3). When t* = 30,000, the two curved particles undergo a certain degree of rotation due to their mutual proximity and the resultant interparticle influences. As the DEP forces acting on both ends of the curved particles are unequal, their mutual interaction prevents them from ultimately rotating to the stable state of 90°. As observed in Figure 3b, the rotation angle gradually decreases after t* = 12,900 before eventually reaching a stable angle. These results demonstrate that curved particles can rotate by 90° to reach a stable state when free from interparticle interactions along the Y-direction. Notably, at an electric field strength of 10 kV/m, face-to-face curved particles exhibit crossed convergence in the vertical orientation while aligning parallel to the electric field. The detailed process of particle motion can be observed in Video S1. This phenomenon suggests that the convergence mode of face-to-face curved particles can be modulated by adjusting the applied electric field strength to an appropriate level.

### 3.2. Shear Modulus

**Figure 4:**
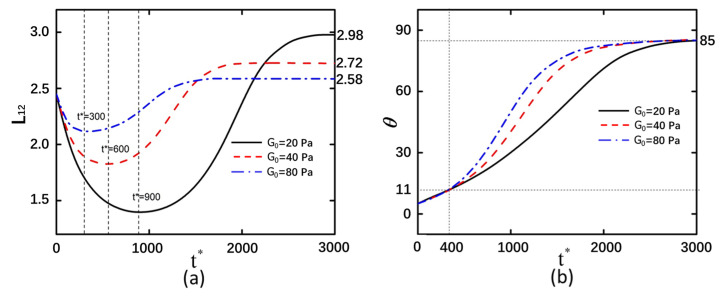
Back-to-back arrangement of curved particles with an electric field strength of 20 kV/m and under shear modulus of 20 Pa, 40 Pa, and 80 Pa. (**a**) Deformation of the left curved particle. (**b**) Rotation angle of the left curved particle.

**Figure 5:**
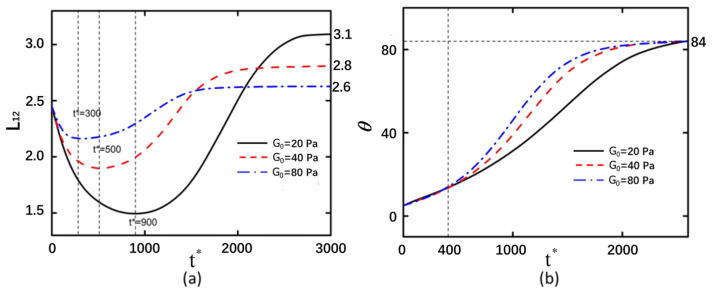
Face-to-face arrangement of curved particles with an electric field strength of 20 kV/m and under shear modulus of 20 Pa, 40 Pa, and 80 Pa. (**a**) Deformation of the left curved particle. (**b**) Rotation angle of the left curved particle.

After altering the shear modulus of the curved particles, the deformation magnitudes and rotational angle trends exhibited consistent behavior in both arrangement configurations (Figure 4 and Figure 5). This indicates that the arrangement configuration of curved particles does not influence the effect of shear modulus changes on deformation behavior. As the shear modulus increases, the compressive and tensile limits achievable by the curved particles decrease. It should be noted that the influence of the shear modulus on the rotation magnitude manifests during the rotational process of curved particles. Prior to the t* = 400 timestep, the rotation magnitude increases gradually, whereas after t* = 400, curved particles with higher elastic moduli exhibit greater slopes in rotation angle variation and faster rates of rotational change. This indicates that particles with larger elastic moduli rotate more readily within equivalent time intervals.

Figure S4 illustrates the pressure distribution of the flow field surrounding the curved particle at t* = 1000. At this temporal point, the flow field pressure between two curved particles with lower elastic moduli is comparatively reduced. Notably, the flow field pressure at one terminal extremity of the curved particle exhibits a marked elevation compared to the opposing terminus. Correlating with the observed counterclockwise rotational phenomenon in face-to-face arranged particles, it can be inferred that the elevated flow field pressure at the curved extremity significantly influences the rotational velocity of the particle. The magnitude of flow field pressure serves as an indicator of the reactive force exerted by the liquid pressure on particle motion, thereby modulating the rotational dynamics of the curved particle. The particles arranged in both back-to-back and face-to-back configurations exhibit counterclockwise rotation. Considering that the displacements of the two curved particles along the X- and Y-axes remain negligible during rotation, it can be inferred that the curved particles rotate approximately about the coordinate origin. Consequently, the pressure in the intermediate flow field has minimal influence on the rotation of the curved particles. A lower shear modulus corresponds to reduced flow field pressure at the extremities of the curved particles, a phenomenon consistent with observations in face-to-back arranged particles. Video S2 illustrates the detailed particle motion process.

### 3.3. Curved Particle Length

**Figure 6:**
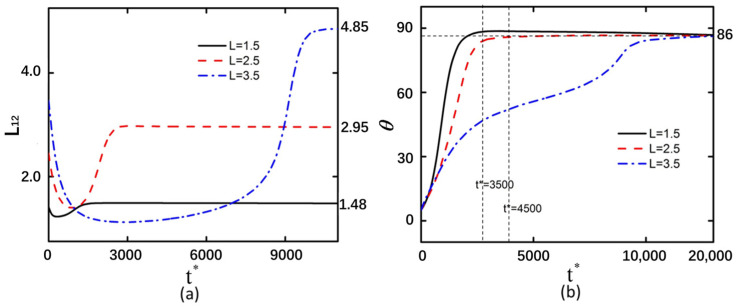
Under an applied electric field of 20 kV/m and with shear modulus *G*_0_ = 20 Pa, back-to-back arranged curved particles demonstrate interparticle endpoint distances of 1.5, 2.5, and 3.5 μm, respectively. (**a**) Deformation profile of the left curved particle. (**b**) Rotation angle of the left curved particle.

**Figure 7:**
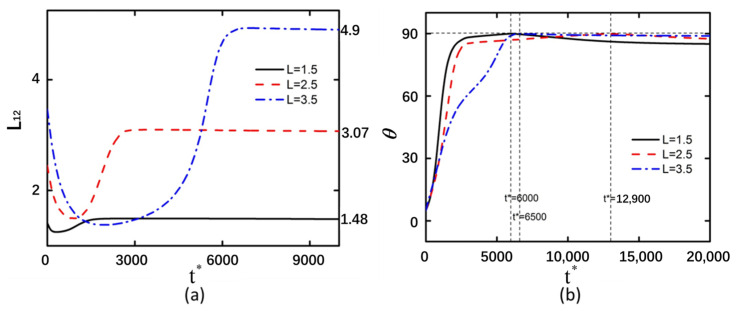
Under an applied electric field of 20 kV/m and with shear modulus *G*_0_ = 20 Pa, face-to-face arranged curved particles demonstrate interparticle endpoint distances of 1.5, 2.5, and 3.5 μm, respectively. (**a**) Deformation profile of the left curved particle. (**b**) Rotation angle of the left curved particle.

The deformation magnitude increases with the enlargement of the curved particle size. However, variations in the dimensions of curved particles exhibit minimal influence on the minimum compression value (Figure 6 and Figure 7). When the curved particles are arranged in a back-to-back configuration, the variations in particle size are 0.02, 0.45, and 1.35, respectively. Conversely, when the curved particles are arranged in a face-to-face configuration, the corresponding variations in particle size are 0.02, 0.57, and 1.4. The back-to-back arranged curved particles with larger sizes exhibit a longer stabilization time compared to face-to-face arranged curved particles of equivalent dimensions. Furthermore, larger curved particle sizes also lead to prolonged compression durations.

The electric field intensity at the two ends of the curved particle exhibits asymmetry, rendering the particle in an unstable state. Concurrently, it was observed that the region of enhanced electric field intensity at the particle’s extremities is predominantly localized on the outer surface. This non-uniform field distribution generates an inwardly directed compressive force toward the central axis, consequently reducing the deformation magnitude of the curved particle (Figure S4). In Figure S4b, the electric field intensity undergoes alterations in the vicinity of the curved particle, while the mutual influence between the two particles remains relatively insignificant. Elevated electric field strengths are observed at the lower vertical electric field region near the extremity of the curved particle and the upper vertical electric field region at its midsection, thereby inducing a rotational tendency of the particle toward 90°. Concurrently, during this temporal phase, the diminished electric field intensity surrounding the curved particle results in the stabilization of its deformation state, with no further modifications occurring. In comparison with Figure S5, when the curved particles are arranged back-to-back, the regions of maximum electric field intensity are localized at the outermost extremities of the particles, exhibiting a concentrated distribution. Conversely, in the face-to-face configuration, although the peak electric field intensity still occurs at the outermost ends of the curved particles, the high-field regions are not concentrated but rather exhibit a fan-shaped distribution. This spatial variation in field distribution consequently affects the compressive forces acting on the particles, resulting in a greater deformation magnitude for back-to-back arranged curved particles compared to their face-to-face counterparts. The compression duration is prolonged when the length of curved particles reaches 3.5. A positive correlation exists between the rotational angle of particles and their deformation magnitude. The deformation of curved particles stabilizes only when their rotation approaches 90°. It should be noted that the electric field intensity at the ends of the curved particles is higher than that in other regions of the particles. However, due to the close proximity between the two curved particles and the mutual influence exerted by each particle, the rotational motion of the particles tends to stabilize.

The electric field intensity distributions of face-to-face and back-to-back arranged curved particles exhibit similar patterns, with high electric field strengths at both ends of the particles leading to particle compression. The asymmetric electric field intensity distribution between the upper and lower ends causes particle rotation (Figure S6). At t* = 6000, the particle is not influenced by another particle along the Y-axis, allowing the curved particle to achieve a rotation angle of 90°. The weak electric field intensity near the particle results in insufficient DEP force to induce deformation. After t* = 6000, the particle’s rotation angle gradually decreases from 90° due to the differential electric field intensities at its two ends, causing minor rotations in the unstable state. Subsequently, influenced by interactions with nearby particles and the equalization of the electric field intensities at both ends of the curved particle, the rotation angle slowly approaches 90° after t* = 6500. At this stage, the DEP force generated by the electric field intensity is insufficient to overcome the particle’s inherent elastic force, preventing further deformation of the curved particle. Consequently, the deformation magnitudes of curved particles of various sizes remain unchanged after t* = 6500. Video S3 illustrates the detailed particle motion process.

### 3.4. The Interparticle Distance

**Figure 8:**
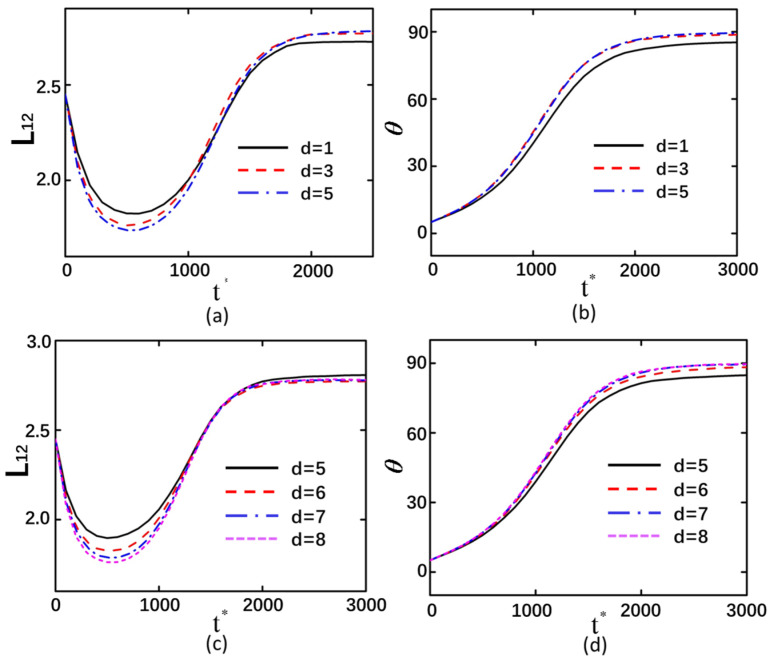
At an applied electric field of 20 kV/m with shear modulus *G*_0_ = 40 Pa. (**a**,**b**) The deformation versus rotation angle profiles for back-to-back configured curved particles, (**c**,**d**) the corresponding curves for left-side face-to-face particle arrangements.

In the back-to-back arrangement of curved particles, the distance between the outer endpoints of the particle centers was selected as the research variable, with values of 1, 3, and 5 μm designated for the separation between the outer endpoints of the two curved particle centers. Conversely, in the face-to-face configuration, the distance between the outer endpoints of the particle centers was likewise chosen as the variable, with specific values of 5, 6, 7, and 8 μm assigned for this parameter. The greater the spacing between curved particles, the smaller the deformation magnitude achievable by the particles. As time progresses and the deformation tends to stabilize, the differences in the final deformation magnitudes become relatively minor. This indicates that larger interparticle spacing leads to greater compression of curved particles during deformation, which is associated with interparticle interactions. When the spacing is small, the influence exerted by adjacent curved particles becomes more pronounced (Figure 8a,c). The variation in the interparticle spacing of curved particles exhibits minimal influence on their rotational displacement. However, particles with larger spacing demonstrate a greater final rotation angle compared to those with smaller spacing, with their rotational orientation tending to approach 90° more closely (Figure 8b,d). This is because closely spaced curved particles are more susceptible to influence from adjacent curved particles along the Y-axis during rotational convergence, resulting in larger rotational displacements for widely spaced particles compared to their closely spaced counterparts during the approach process. The detailed process of particle motion can be observed in Video S4.

### 3.5. The Arrangement Pattern of Curved Particles

**Figure 9:**
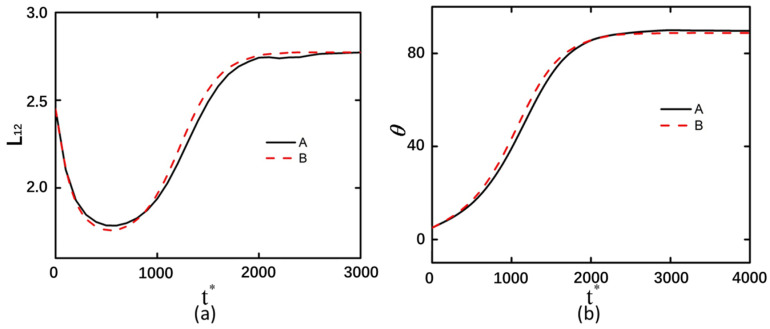
Under an electric field intensity of 20 kV/m, with shear modulus of *G*_0_ = 40 Pa and an interparticle spacing of 5 μm between two curved particles: (**a**) deformation profile of the two curved particles; (**b**) rotational angle profile of the two curved particles. A denotes the left curved particle, while B represents the right curved particle.

In practice, curved particles may exhibit non-back-to-back and non-face-to-face arrangements; therefore, the particle alignment configuration has been adjusted accordingly. In the study of back-to-back and face-to-face curvilinear particle arrangements, the particles are symmetrically configured. Consequently, the analysis of one side of the particle array is sufficient to derive the results for the opposite side. The co-directional curved particles are not symmetrically distributed (with both curved particles bending in the same direction); thus, it is necessary to first analyze the deformation and rotation angles of the two curved particles. The variation trends of deformation and rotation for the two curved particles aligned in the same direction exhibit good agreement. However, the minimum achievable deformation of the right curved particle is smaller than that of the left one, indicating greater deformation magnitude in the right particle (Figure 9a). The rotational angle likewise exhibits a more rapid variation on the right-side particles (Figure 9b), indicating that the particles on the right side experience a stronger electric field force and demonstrate more pronounced motion characteristics. When the two curved particles are no longer symmetrically distributed, the electric field forces acting on them become unequal, consequently leading to divergent motion behaviors between the particles. At t* = 1000, the right-hand particle displays markedly greater rotation than the left particle (Figure S7). Post t* = 1000, curved particle A exhibits slower deformation evolution and reduced angular velocity compared to its right counterpart (particle B).

**Figure 10:**
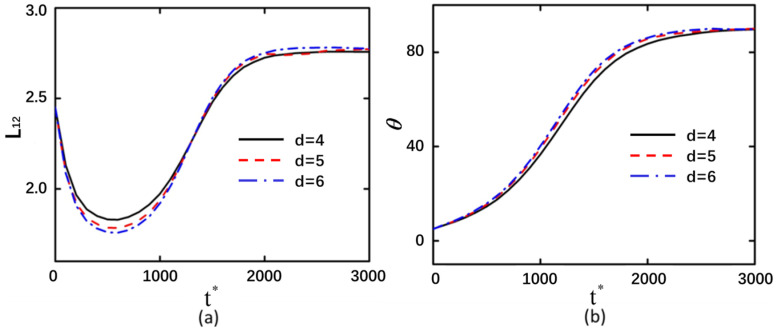
The electric field intensity is 20 kV/m, with shear modulus of *G*_0_ = 40 Pa, and the spacings between the two curved particles are 4, 5, and 6 μm, respectively. (**a**) The deformation of the left curved particle. (**b**) The rotation angle of the left curved particle.

When the interparticle distance is altered, a greater distance results in higher compressive stress on the curved particles, yet the ultimate deformation tends to converge, as shown in Figure 10. The variation in the curved particle spacing exhibits minimal influence on the rotational angle change. This finding is consistent with the trend observed in Figure 8. The detailed process of particle motion can be observed in Video S5.

## 4. Conclusions

This study investigates the DEP interactions and motion of curved particles suspended in a solution under the influence of a direct current electric field. The presence of two curved particles transforms the uniform electric field into a non-uniform one, inducing DEP in the curved particles. Upon exposure to the DC electric field, the particles undergo rotation, deformation, and mutual attraction through DEP, eventually forming a stable alignment parallel to the applied DC electric field direction. Higher electric field intensities result in more pronounced particle deformation and faster rotational angle changes. When the curved particles are arranged asymmetrically, their motion follows trends similar to face-to-face and back-to-back configurations; however, they ultimately align with their openings oriented upward, forming chains parallel to the electric field lines. Spherical or rod-shaped particles, due to their completely symmetrical structure, exhibit nearly symmetrical forms during deformation and motion. In contrast, curved particles bend and rotate under the influence of electric field forces without significant changes in their shape. These results provide certain theoretical guidance for the manipulation of cells, droplets, and other entities based on DEP technology, such as cell separation or multi-droplet manipulation.

## Data Availability

The raw data supporting the conclusions of this article will be made available by the authors on request.

## Supplementary Materials

The following supporting information can be downloaded at: https://www.mdpi.com/article/10.3390/mi16050596/s1, Figure S1. Model Validation; Figure S2. The curved particles are arranged back-to-back with shear modulus of 20 Pa. (a–c) correspond to *t** = 0, (d–f) correspond to *t** = 2000, while (g,h,i) represent *t** = 20,000, *t** = 30,000, and *t** = 20,000, respectively. (a,d,g) display the electric field intensity distribution of arcuate particles at 5 kV/m, (b,e,h) at 10 kV/m, and (c,f,i) at 20 kV/m; Figure S3. The curved particles are arranged in a face-to-face configuration with shear modulus of 20 Pa. (a–c) correspond to time *t** = 0; (d–f) represent time *t** = 12,900; while (g,h,i) depict times *t** = 200,000, *t** = 200,000, and *t** = 30,000, respectively. The electric field intensity distributions of arcuate particles are shown under different field intensities: (a,d,g) at 5 kV/m; (b,e,h) at 10 kV/m; and (c,f,i) at 20 kV/m; Figure S4. The pressure distribution of the flow field around the particle at time *t** = 1000: (a,b,c) correspond to face-to-face particle configurations with elastic moduli of *G*_0_ = 20 Pa, *G*_0_ = 40 Pa, and *G*_0_ = 80 Pa, respectively; (d,e,f) represent back-to-back particle arrangements with elastic moduli of *G*_0_ = 20 Pa, *G*_0_ = 40 Pa, and *G*_0_ = 80 Pa, respectively; Figure S5. The particles are arranged back-to-back, and the distribution of electric field intensity around the particles is illustrated under the following conditions: an applied electric field strength of 20 kV/m, an elastic modulus of *G*_0_ = 20 Pa, and arc-shaped particle endpoint separations of (a,b) 1.5, (c,d) 2.5, and (e,f) 3.5. The corresponding time instances are (a,c,e) *t** = 100, (b) *t** = 3500, (d) *t** = 4500, and (f) *t** = 12,900; Figure S6. The distribution of electric field intensity around particles arranged in a face-to-face configuration is presented under the following conditions: an applied electric field strength of 20 kV/m, an elastic modulus of *G*_0_ = 20 Pa, and interparticle endpoint distances of (a,b) 1.5, (c,d) 2.5, and (e,f) 3.5. The corresponding dimensionless time instants are (a,c,e) *t** = 100, (b) *t** = 6000, (d) *t** = 6500, and (f) *t** = 12,900; Figure S7. The electric field intensity is 20 kV/m, the elastic modulus is *G*_0_ = 40 Pa, and the separation between the two curved particles is 5 μm. The distribution of electric field intensity near the curved particles is illustrated as follows: (a) *t** = 0, (b) *t** = 1000, (c) *t** = 20,000. Video S1: particle motion under varying electric field intensities. Video S2: particle motion with different shear moduli. Video S3: particle motion across varying particle length. Video S4: particle motion at different interparticle distances. Video S5: particle motion in diverse arrangements.

## Abbreviations

The following abbreviations are used in this manuscript:

DEP	dielectrophoresis
EDL	electrical double layer
DC	direct current
ALE	arbitrary Lagrangian–Eulerian
