# Supplementary material for: The Dielectrophoretic Interactions of Curved Particles in a DC Electric Field
Source: Micromachines (Basel). 2025 May 20;16(5):596. doi: 10.3390/mi16050596 (PMC12113738; doi:10.3390/mi16050596)
Supplement: Supplementary file 1 [file micromachines-16-00596-s001.zip › Supplementary Materials.pdf]

The Dielectrophoretic Interactions of Curved Particles in a DC Electric Field

Zhiwei Huang *, Tong Zhang * , Jing Feng and Yage Wang

Mechanical and Electrical Engineering College, Guangdong University of Science and Technology, Dongguan 523668, China
* Correspondence: huangzhiwei2@gdust.edu.cn (Z.H.); zhangtong@gdust.edu.cn (T.Z.);
Tel.: +86-769-8802-7157 (Z.H. & T.Z.)

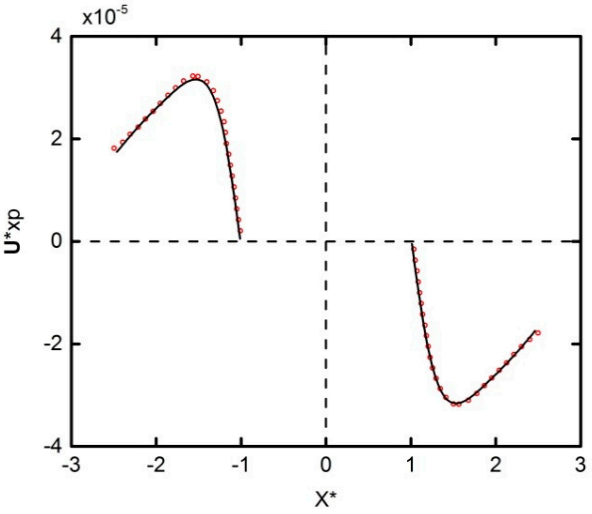

Figure S1. Model Validation.

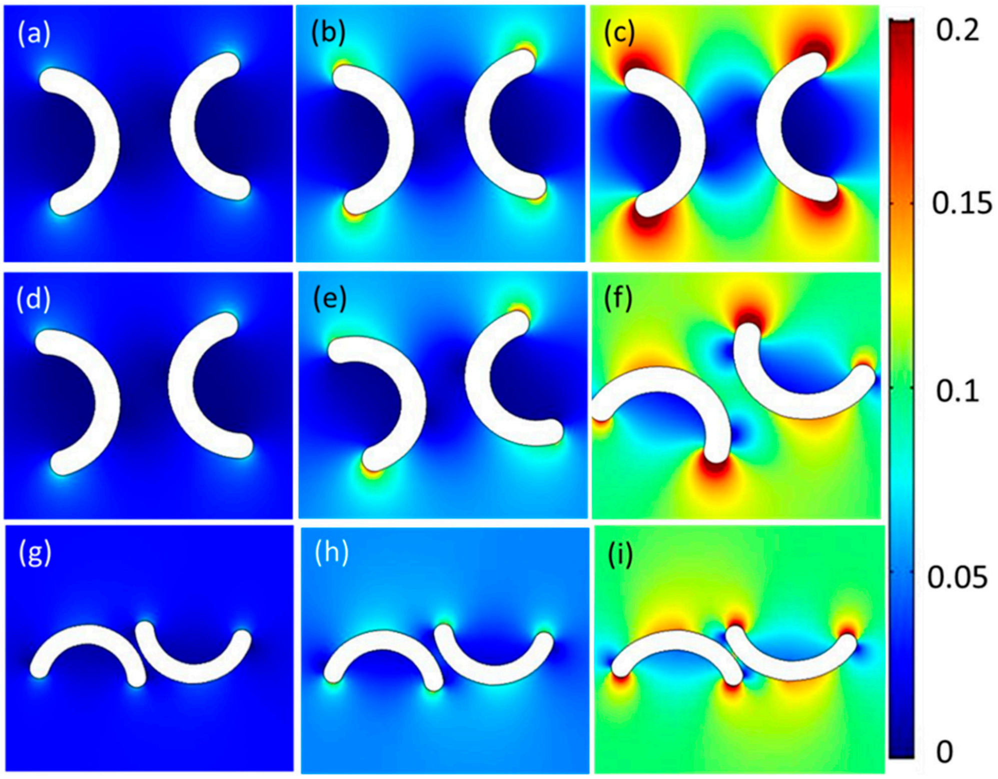

Figure S2. The curved particles are arranged back-to-back with shear modulus of 20 Pa. (a) (b) (c) correspond to t*=0, (d) (e) (f) correspond to t*=2000, while (g) (h) (i) represent t* =20,000, t*=30,000, and t*=20,000, respectively. (a) (d) (g) display the electric field intensity distribution of arcuate particles at 5 kV/m, (b) (e) (h) at 10 kV/m, and (c) (f) (i) at 20 kV/m.

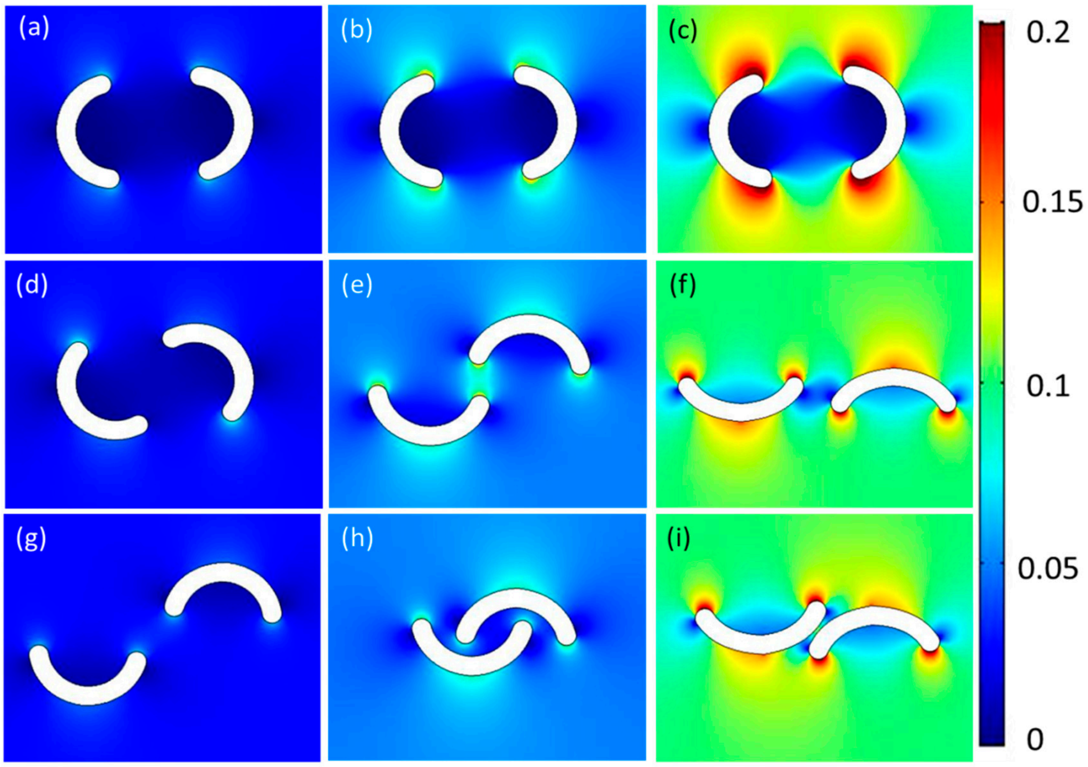

Figure S3. The curved particles are arranged in a face-to-face configuration with shear modulus of 20 Pa. (a), (b), and (c) correspond to time t^*=0; (d), (e), and (f) represent time t^*=12,900; while (g), (h), and (i) depict times t^*=200,000, t^*=200,000, and t^*=30,000, respectively. The electric field intensity distributions of arcuate particles are shown under different field intensities: (a), (d), and (g) at 5 kV/m; (b), (e), and (h) at 10 kV/m; and (c), (f), and (i) at 20 kV/m.

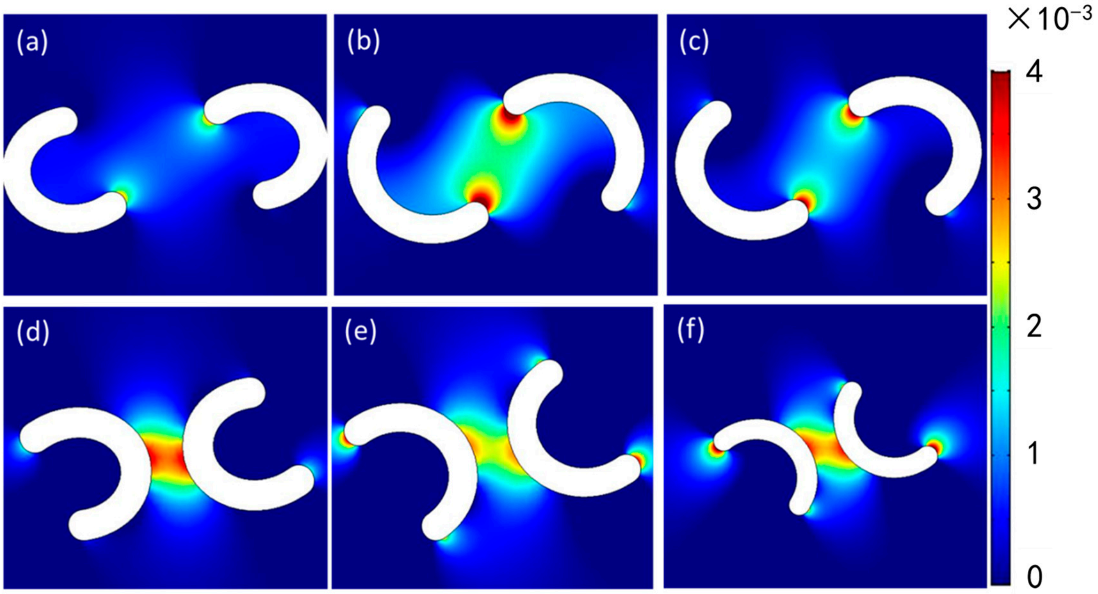

Figure S4. The pressure distribution of the flow field around the particle at time t^*=1000: (a)(b)(c) correspond to face-to-face particle configurations with elastic moduli of G_0=20Pa, G_0=40Pa, and G_0=80Pa respectively; (d)(e)(f) represent back-to-back particle arrangements with elastic moduli of G_0=20Pa, G_0=40Pa, and G_0=80Pa respectively.

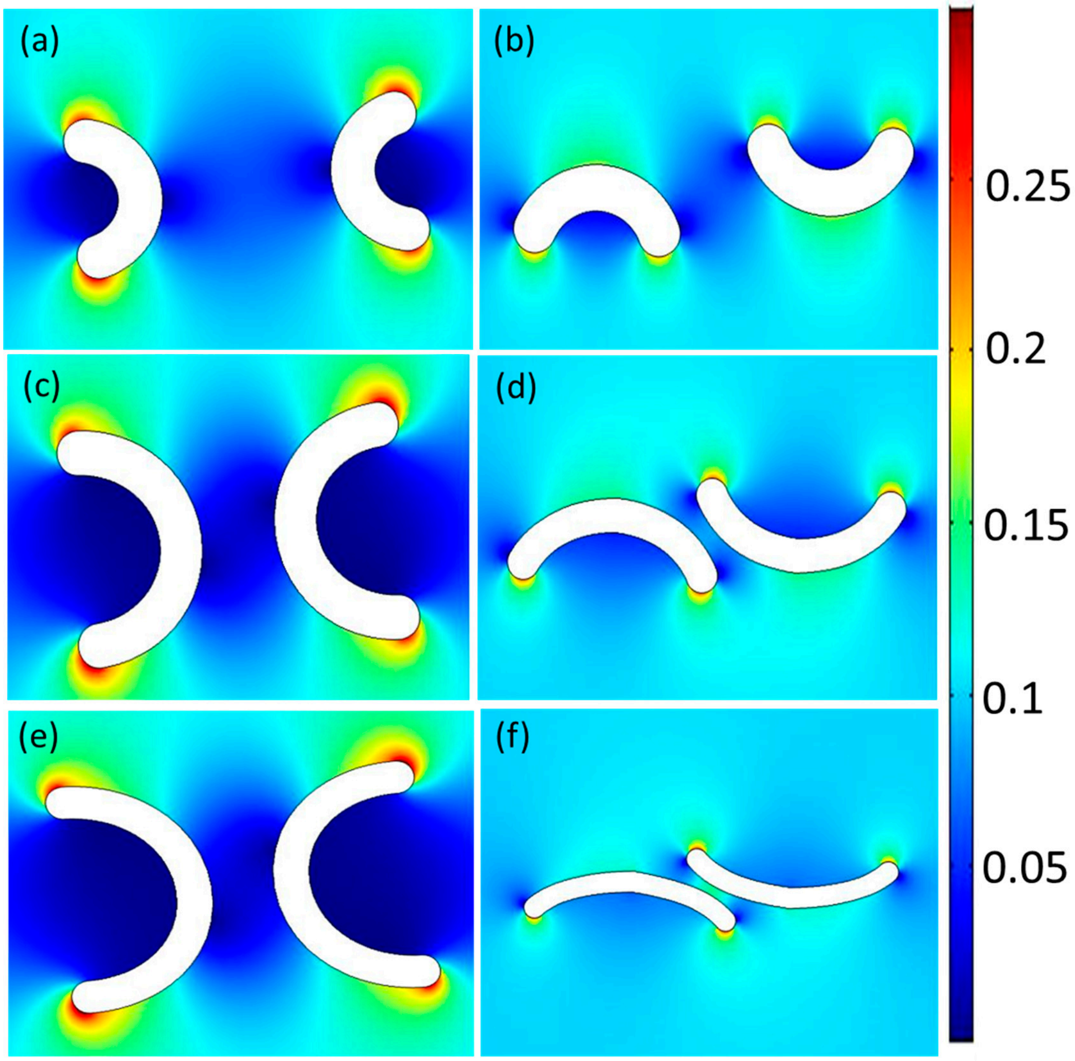

Figure S5. The particles are arranged back-to-back, and the distribution of electric field intensity around the particles is illustrated under the following conditions: an applied electric field strength of 20 kV/m, an elastic modulus of G_0 = 20 Pa, and arc-shaped particle endpoint separations of (a)(b) 1.5, (c)(d) 2.5, and (e)(f) 3.5. The corresponding time instances are (a)(c)(e) t^* = 100, (b) t^* = 3500, (d) t^* = 4500, and (f) t^* = 12900.

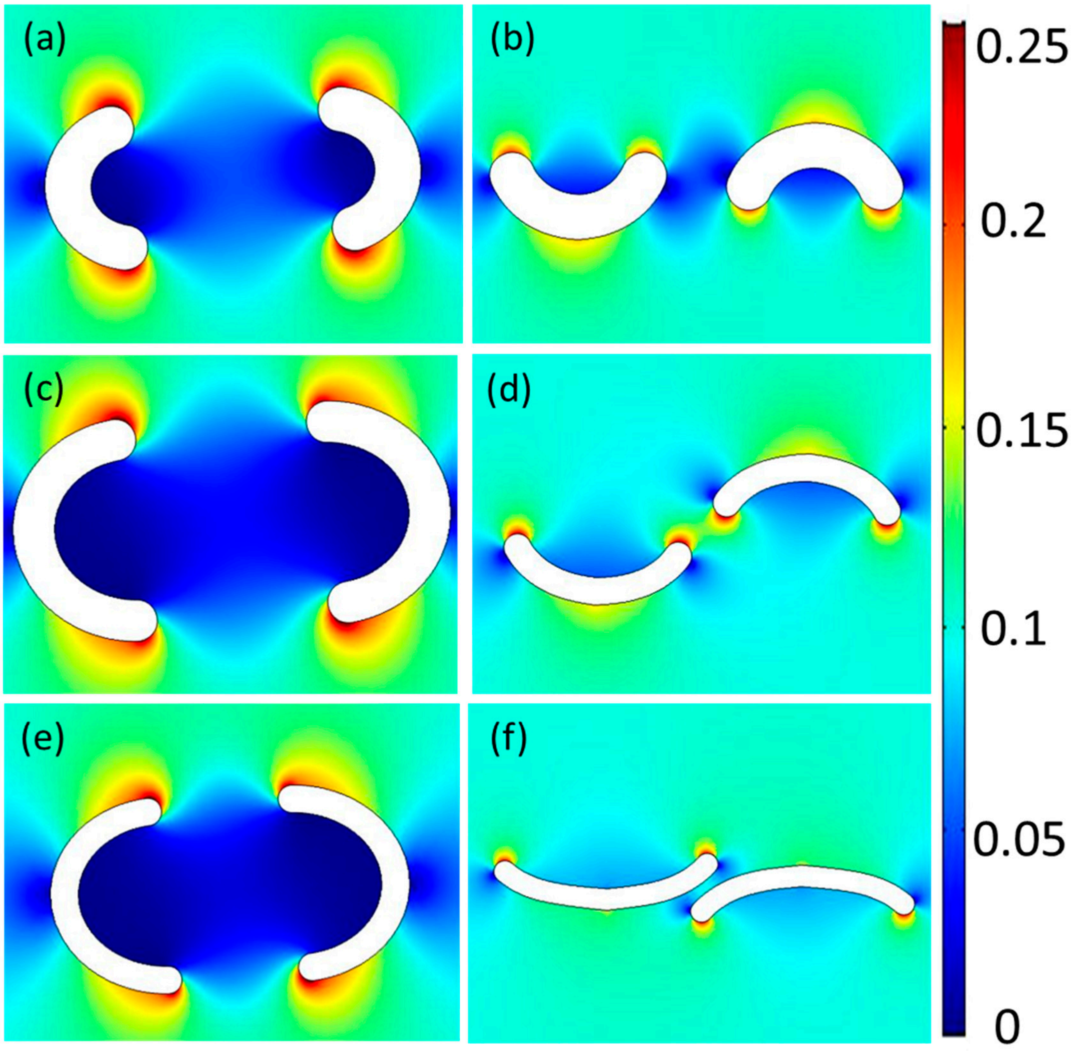

Figure S6. The distribution of electric field intensity around particles arranged in a face-to-face configuration is presented under the following conditions: an applied electric field strength of 20 kV/m, an elastic modulus of G_0 = 20 Pa, and interparticle endpoint distances of (a)(b) 1.5, (c)(d) 2.5, and (e)(f) 3.5. The corresponding dimensionless time instants are: (a)(c)(e) t^* = 100, (b) t^* = 6000, (d) t^* = 6500, and (f) t^* = 12900.

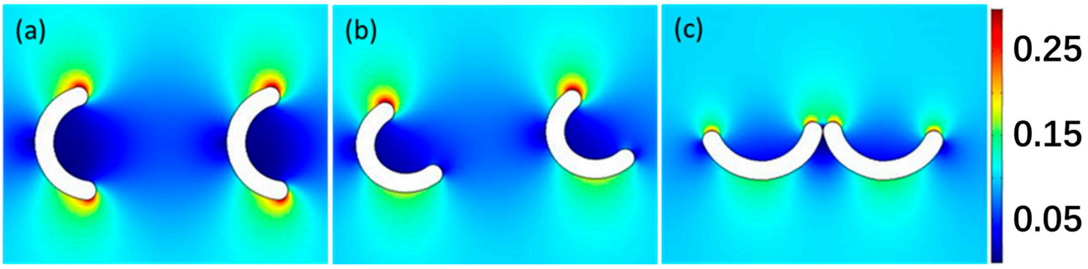

Figrue S7. The electric field intensity is 20 kV/m, the elastic modulus is G_0 = 40 Pa, and the separation between the two curved particles is 5 μm. The distribution of electric field intensity near the curved particles is illustrated as follows: (a) t^* = 0, (b) t^* = 1000, (c) t^* = 20000.
