# Supplementary figures and images for: The Dielectrophoretic Interactions of Curved Particles in a DC Electric Field
Source: Micromachines (Basel). 2025 May 20;16(5):596. doi: 10.3390/mi16050596 (PMC12113738; doi:10.3390/mi16050596)

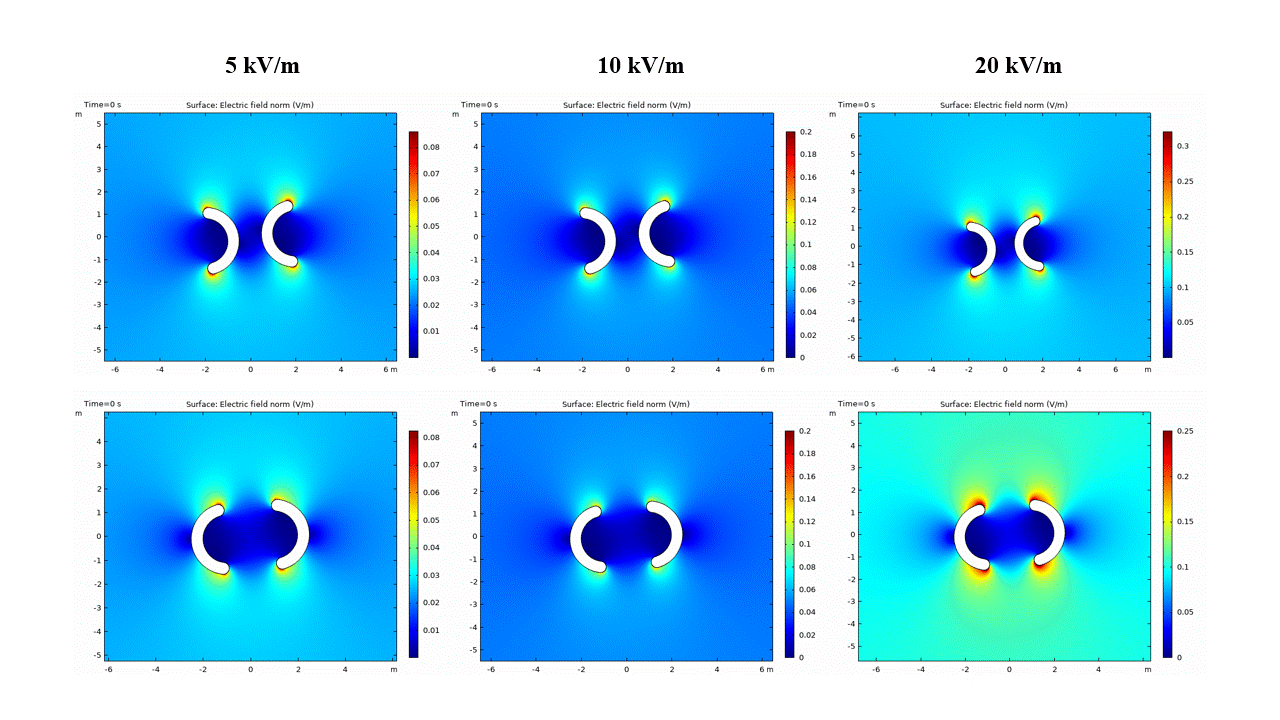

Supplement: Supplementary file 1 [file micromachines-16-00596-s001.zip › Video/Video S1.gif]

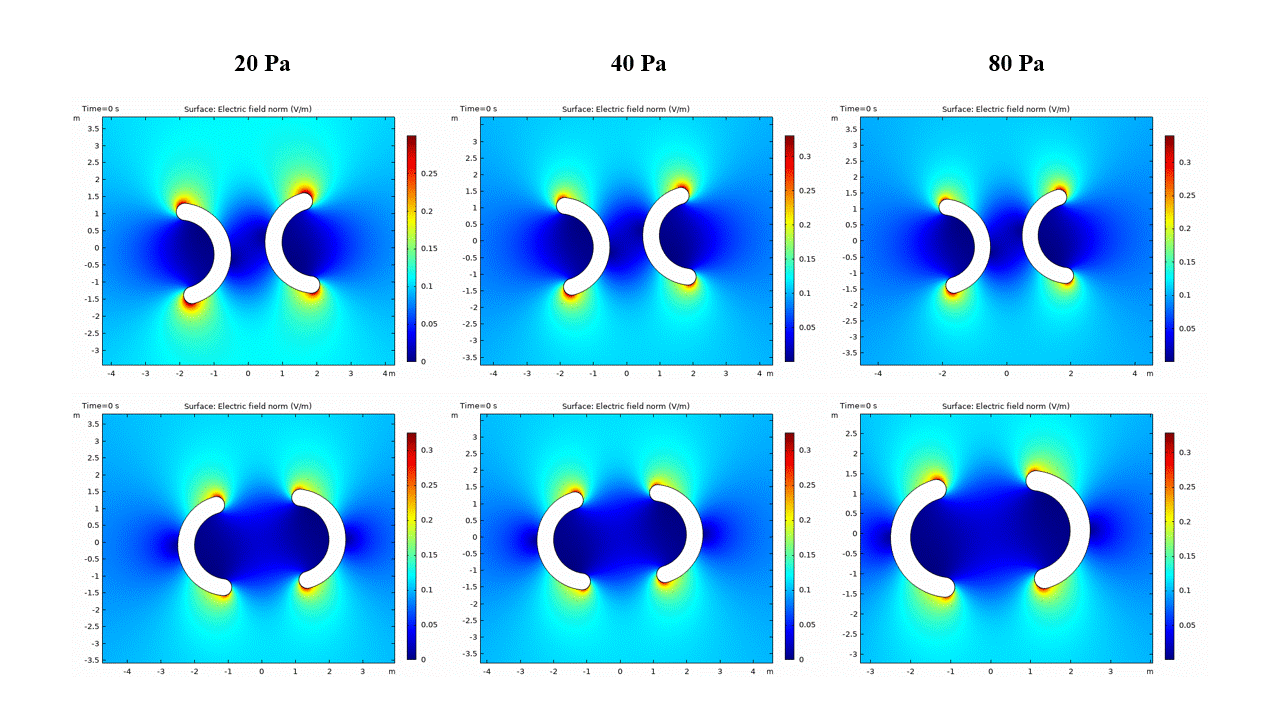

Supplement: Supplementary file 1 [file micromachines-16-00596-s001.zip › Video/Video S2.gif]

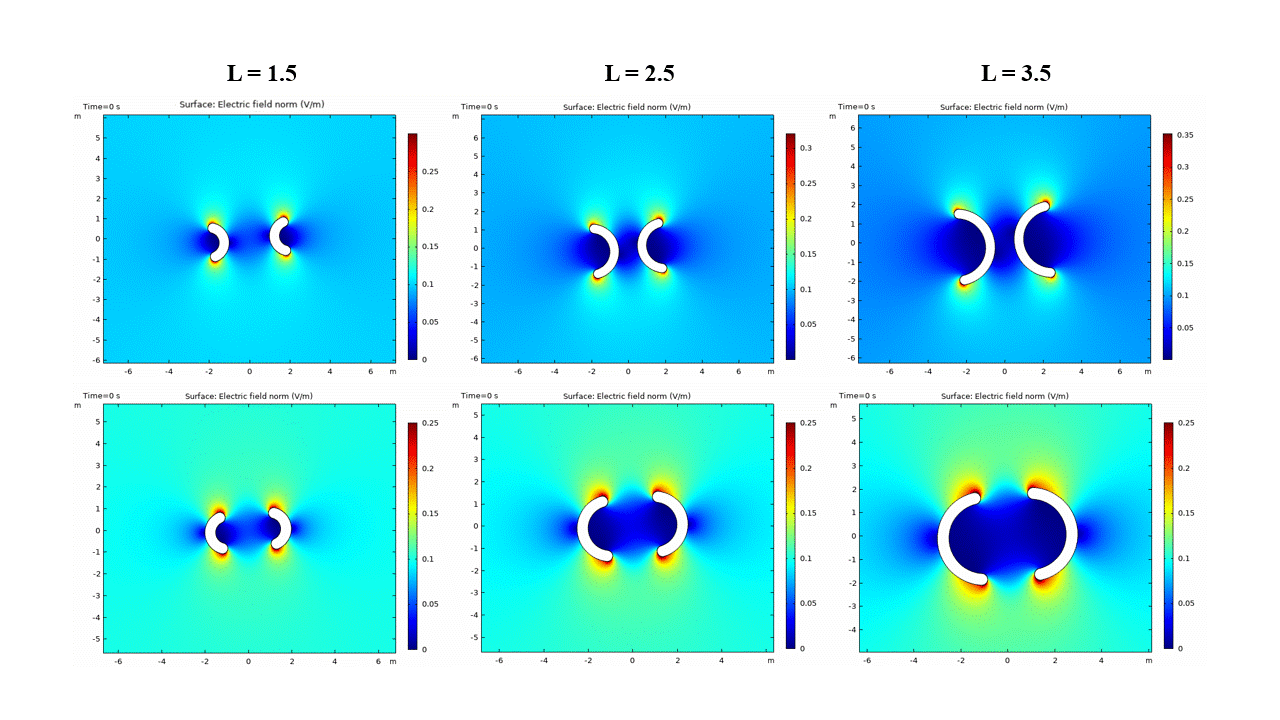

Supplement: Supplementary file 1 [file micromachines-16-00596-s001.zip › Video/Video S3.gif]

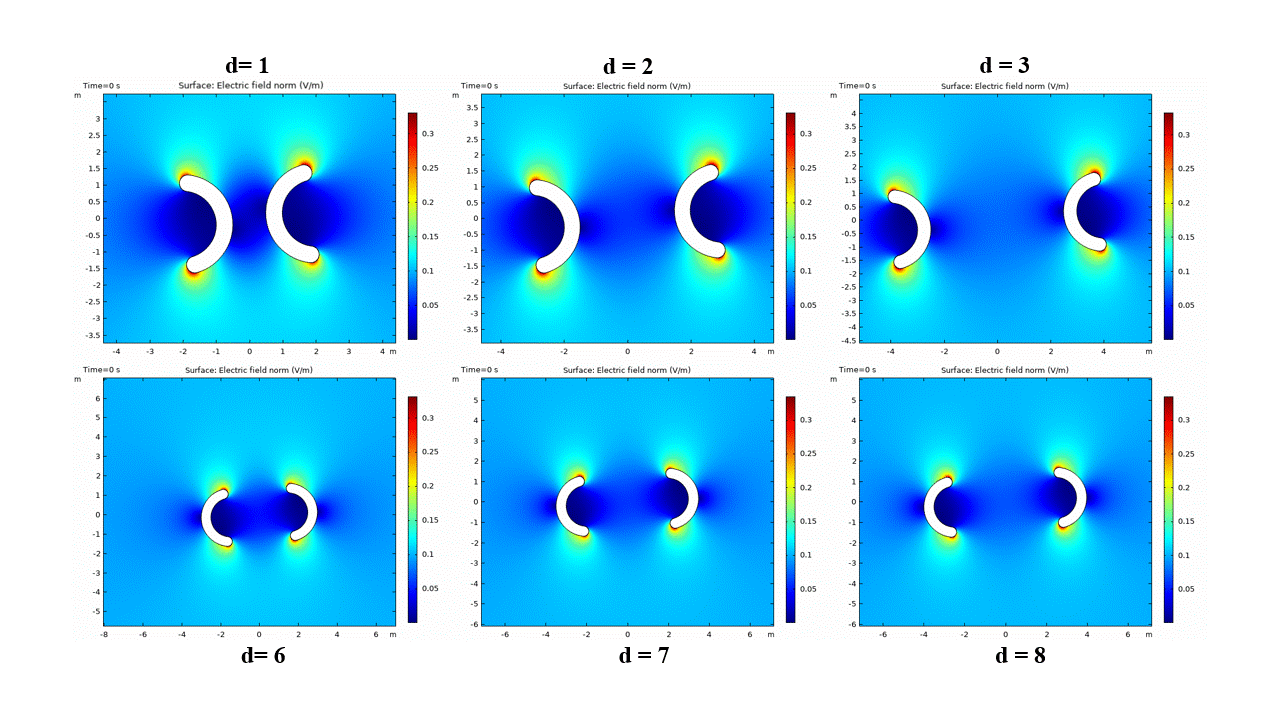

Supplement: Supplementary file 1 [file micromachines-16-00596-s001.zip › Video/Video S4.gif]

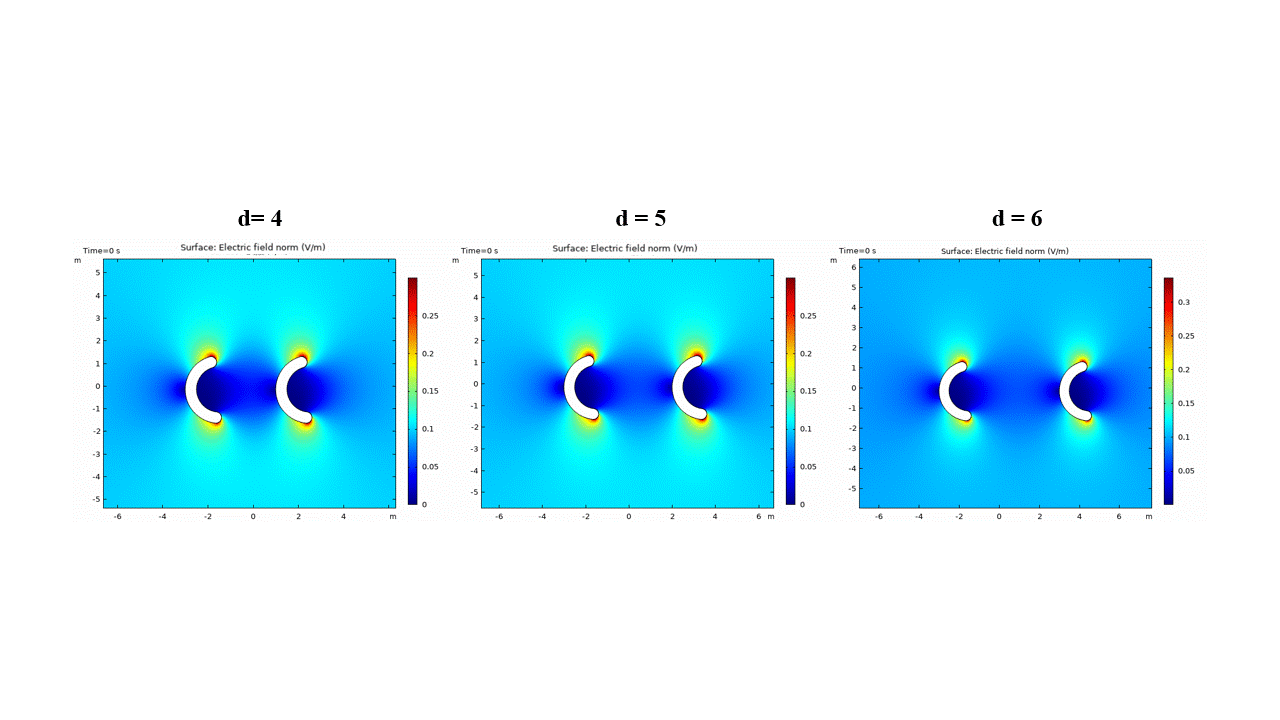

Supplement: Supplementary file 1 [file micromachines-16-00596-s001.zip › Video/Video S5.gif]
